# Acute Kidney Failure and Myocarditis Triggered by Magic Mushroom Toxicity in a Patient With Prior Cocaine Exposure

**DOI:** 10.7759/cureus.96718

**Published:** 2025-11-12

**Authors:** Aung Phyo Oo, Sarah Tseu, Arvind Ponnusamy

**Affiliations:** 1 Nephrology, Royal Preston Hospital, Preston, GBR; 2 Nephrology, Lancashire Teaching Hospitals NHS Foundation Trust, Preston, GBR; 3 Nephrology, Lancashire Teaching Hospitals NHS Foundation Trust, Royal Preston Hospital, Preston, GBR

**Keywords:** acute tubular necrosis, dialysis, magic mushroom, myocarditis, thrombosis

## Abstract

Magic mushroom poisoning can be associated with acute kidney injury (AKI), primarily due to ischemic acute tubular necrosis (ATN). Additionally, the use of cocaine can lead to both venous and arterial thrombosis through its vasoconstrictive and prothrombotic effects. In this article, a middle-aged gentleman with a previous history of cocaine use was admitted with severe anuric AKI requiring dialysis after magic mushroom poisoning. He developed supraventricular tachycardia (SVT) with significantly raised troponin T and severe left ventricular dysfunction, which was thought to be due to myocarditis induced by magic mushrooms. In addition, imaging revealed extensive thrombosis in multiple blood vessels, including the abdominal aorta, superior mesenteric artery (SMA), and bilateral iliac arteries, resulting in right kidney infarction and a pulmonary embolism in the right lower lobe. There is also a possibility that psilocybin-containing magic mushrooms may induce vasoconstriction, which could theoretically contribute to thrombotic events. However, direct evidence linking psilocybin to thrombosis remains limited.

## Introduction

Magic (psilocybin-containing) mushrooms are increasingly used recreationally and in some controlled therapeutic contexts because of their psychoactive effects. Most published series and toxicology reviews indicate that adverse events from psilocybin ingestion are typically neuropsychiatric, transient, and self-limited, with serious systemic toxicity regarded as uncommon [1].

Nevertheless, rare but important reports have documented acute kidney injury (AKI) following ingestion of hallucinogenic mushrooms. Some case reports suggest that magic mushroom-associated renal injury can be severe and occasionally requires renal replacement therapy [1].

Accordingly, we report a case of symptomatic AKI requiring dialysis temporally related to the ingestion of recreational "magic" mushrooms (a species commonly associated with psilocybin), with emphasis on the clinical course, investigative findings, management, and potential mechanisms.

## Case presentation

A middle-aged gentleman with type 2 diabetes mellitus (T2DM), hypertension, peripheral vascular disease, and a prior non-ST elevation myocardial infarction (NSTEMI) was transferred from a district hospital to our renal tertiary hospital for further evaluation and management of AKI following suspected magic mushroom toxicity.

The patient presented with general malaise, nausea, abdominal pain, persistent retching, diarrhea, sweating, tachycardia, and visual hallucinations approximately five hours after ingesting magic mushrooms in combination with alcohol and lysergic acid diethylamide (LSD), and he remained unwell for several days thereafter. Upon assessment, it was noted that he had been oligoanuric for approximately four days prior to admission to his local hospital. A routine toxicology panel was obtained prior to the patient’s transfer to our hospital, yielding negative results. His medical history was also notable for prior cocaine use, although he reported no recent use at the time of this presentation. He also denied the use of any regular medications, including herbal or traditional remedies. During his admission, the patient remained anuric despite improvement in his presenting symptoms. Hemodialysis was continued due to ongoing anuria and significant electrolyte disturbances.

**Figure 1:**
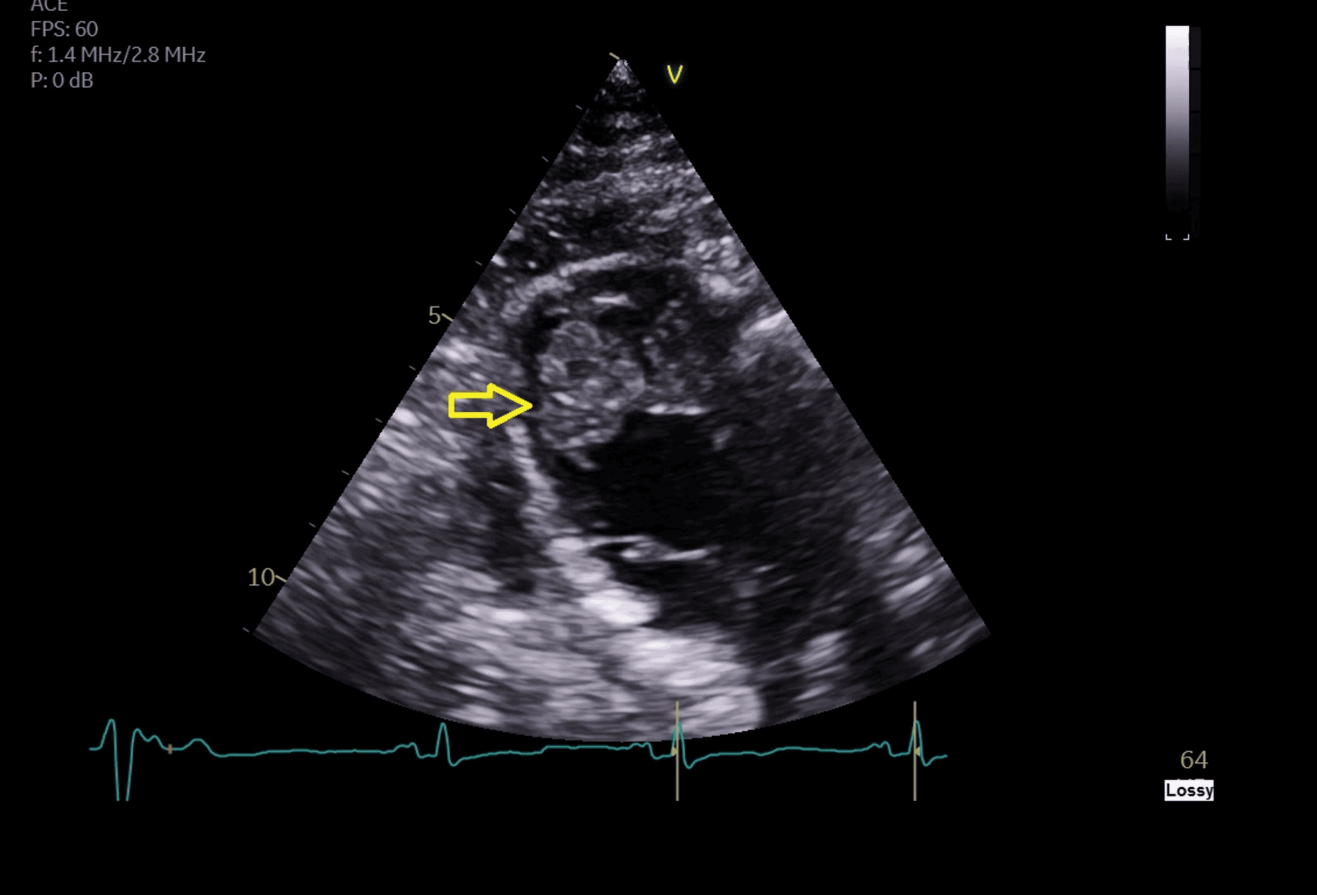
Echocardiogram showing a mural thrombus in the left ventricle Transthoracic echocardiogram (parasternal short-axis view at the mid-ventricular level) showed a mural thrombus (yellow arrow) attached to the left ventricular endocardium. The thrombus appears echogenic and distinct from the adjacent myocardium.

On admission, initial laboratory investigations revealed mildly elevated C-reactive protein (CRP) and white blood cell (WBC) count, which were already being addressed with oral co-amoxiclav for a suspected chest infection (Table 1). Creatine kinase (CK) levels were within the normal reference range and not of clinical concern. Deranged renal and liver function tests were consistent with toxin-related injury, likely related to magic mushroom ingestion, demonstrating a transaminitis pattern indicative of hepatitis. A comprehensive renal immunology panel was performed, including antinuclear antibody (ANA), anti-double-stranded DNA (anti-dsDNA), complements, antineutrophil cytoplasmic antibody (ANCA), and connective tissue disease screen, which returned negative. Transthoracic echocardiography identified heart failure with reduced ejection fraction (an estimated ejection fraction of 35%) with a left ventricular mural thrombus (Figure 1), prompting initiation of therapeutic anticoagulation.

**Table 1:** Comparison of blood investigations on admission and on June 29, 2024 Laboratory parameters on admission and during hospital stay showed progressive elevation of inflammatory markers (C-reactive protein and white cell count), worsening renal function (rising urea and creatinine with declining estimated glomerular filtration rate), and marked troponin elevation. Liver enzymes were initially elevated with subsequent improvement, while electrolyte abnormalities included persistent hyperkalemia and hyperphosphatemia.

Laboratory Tests	Results on June 19, 2024 (on admission)	Results on June 29, 2024	Normal Range
C-reactive protein	112.8	228.4	0.0-5.0 mg/L
White cell count	12.47	18.91	4.00-11.00 x 10^9^/L
Haemoglobin	131	113	130-180 g/L
Platelet	140	322	140-440 x 10^9^/L
Mean corpuscular volume	85.5	83.5	82.0-98.0 fL
Neutrophils	9.43	16.69	1.60-7.50 x 10^9^/L
Lymphocytes	1.74	1.40	1.00-4.00 x 10^9^/L
Sodium	134	134	133-146 mmol/L
Potassium	5.3	6.2	3.5-5.3 mmol/L
Urea	26.3	41.5	2.5-7.8 mmol/L
Creatinine	482	844	59-104 µmol/L
Troponin	-	16,774	5-14 ng/L
Estimated glomerular filtration rate	10	5	
Total bilirubin	20	18	0-21 µmol/L
Alkaline phosphatase	185	106	30-130 U/L
Gamma-glutamyl transferase	107	64	0-71 U/L
Total protein	56	70	60-80 g/L
Albumin	31	33	35-50 g/L
Alanine aminotransferase	692	172	0-50 U/L
Correct calcium	2.25	2.30	2.20-2.60 mmol/L
Phosphate	2.02	3.91	0.80-1.50 mmol/L

**Figure 2:**
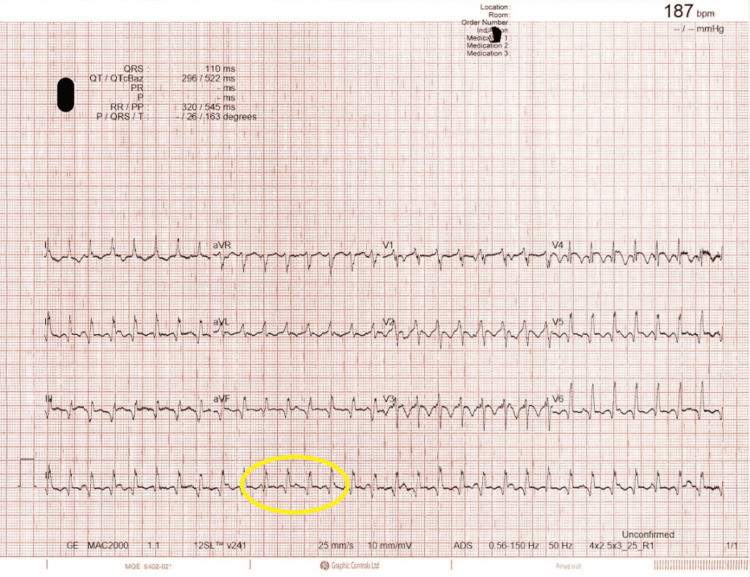
ECG showing supraventricular tachycardia (SVT) with heart rate (HR) of 187 bpm Twelve-lead electrocardiogram showed a regular narrow complex tachycardia (yellow circle) at a rate of approximately 187 beats per minute, consistent with supraventricular tachycardia.

On the fifth day of hospitalization, the patient developed supraventricular tachycardia (SVT) with a heart rate ranging from 170 to 180 beats per minute (Figure 2) and associated hypotension (systolic blood pressure around 90 mmHg). A dose of bisoprolol was administered prior to adenosine. Afterward, three incremental doses of adenosine were administered to achieve AV nodal blockade for SVT, but this was unsuccessful. An amiodarone loading dose followed by a maintenance infusion was given, resulting in successful rhythm control. The patient was then transferred to the Coronary Care Unit for close monitoring.

In light of the elevated troponin T levels, severe left ventricular systolic dysfunction, arrhythmias, and absence of ischemic changes on electrocardiogram or new infarction on imaging, a diagnosis of severe myocarditis was considered. Based on the temporal relationship with toxic ingestion, magic mushroom-induced myocarditis was deemed the most likely etiology. We attempted to proceed with cardiac magnetic resonance imaging (MRI) to look for subendocardial fibrosis or edema; however, the patient’s clinical condition precluded safe transfer to a facility equipped to perform the scan.

**Figure 3:**
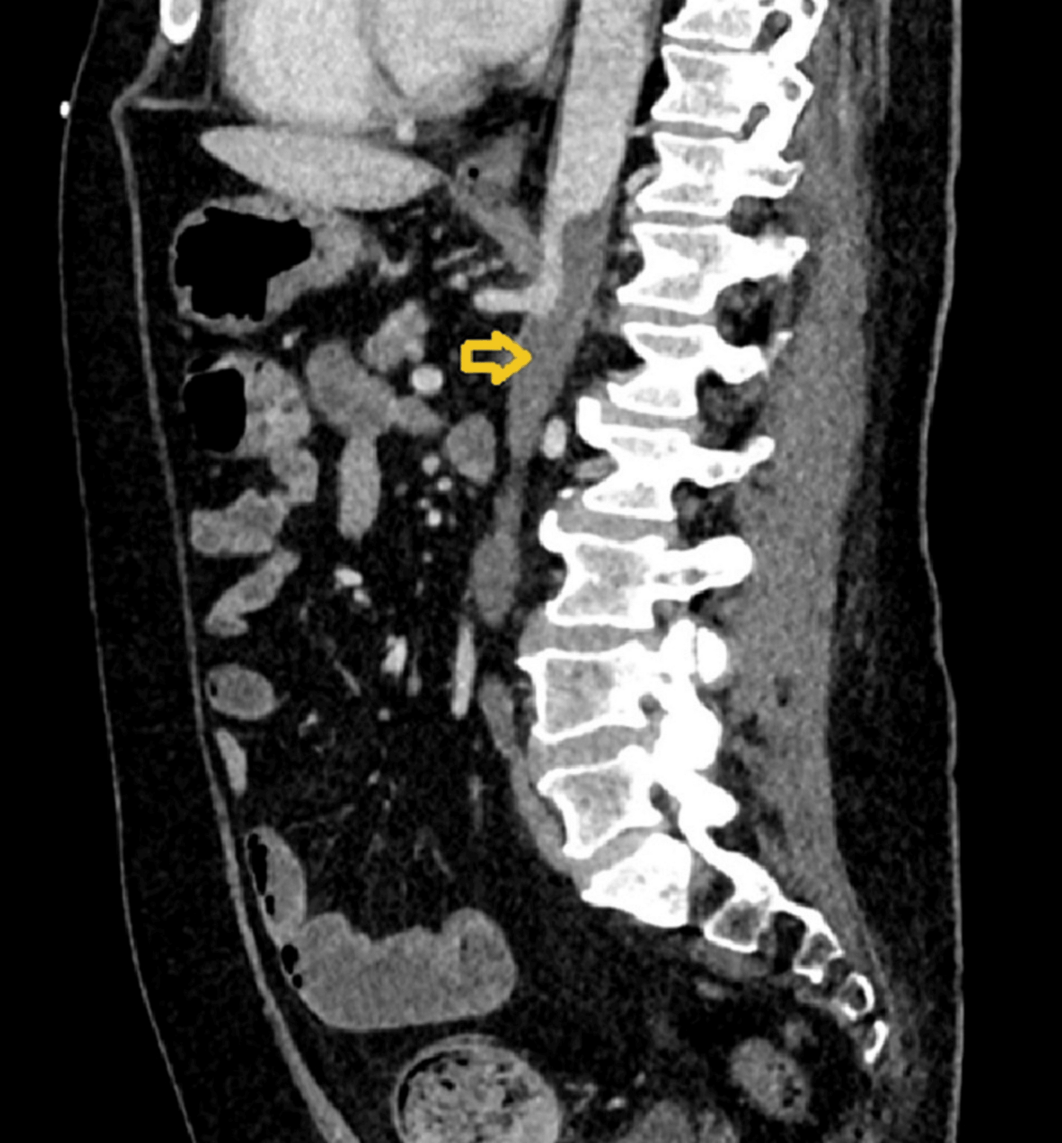
Extensive thrombosis involving multiple vascular territories Contrast-enhanced computed tomography (sagittal view) of the abdomen demonstrated extensive thrombosis involving multiple vascular territories, including the abdominal aorta distal to the coeliac axis, with thrombus extension into the internal and external iliac arteries (yellow arrow).

**Figure 4:**
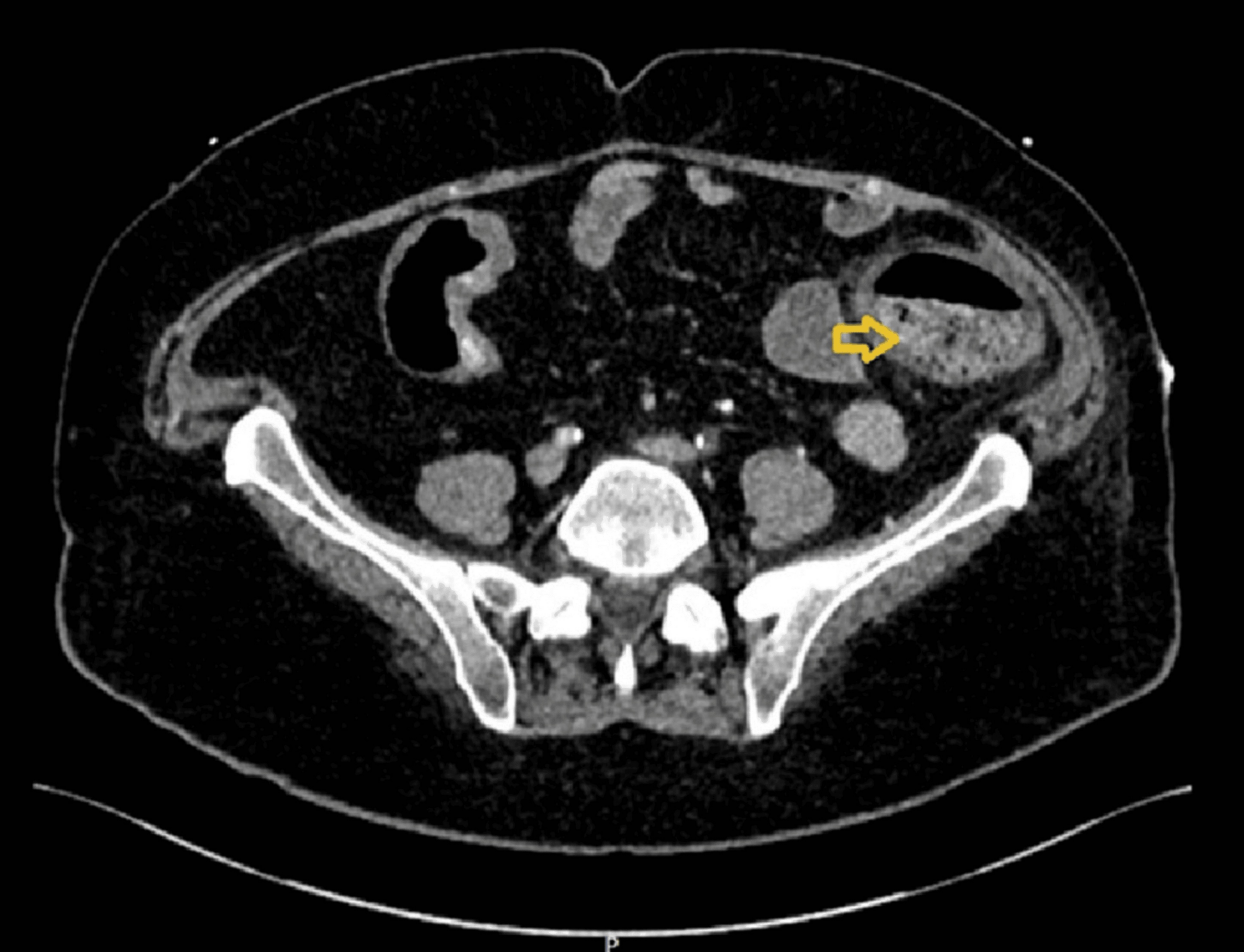
Vascular watershed ischemia in the descending colon The above CT scan (yellow arrow) revealed that the descending colon in a vascular watershed zone showed thickening and peri-colonic fat stranding in keeping with acute ischemia.

**Figure 5:**
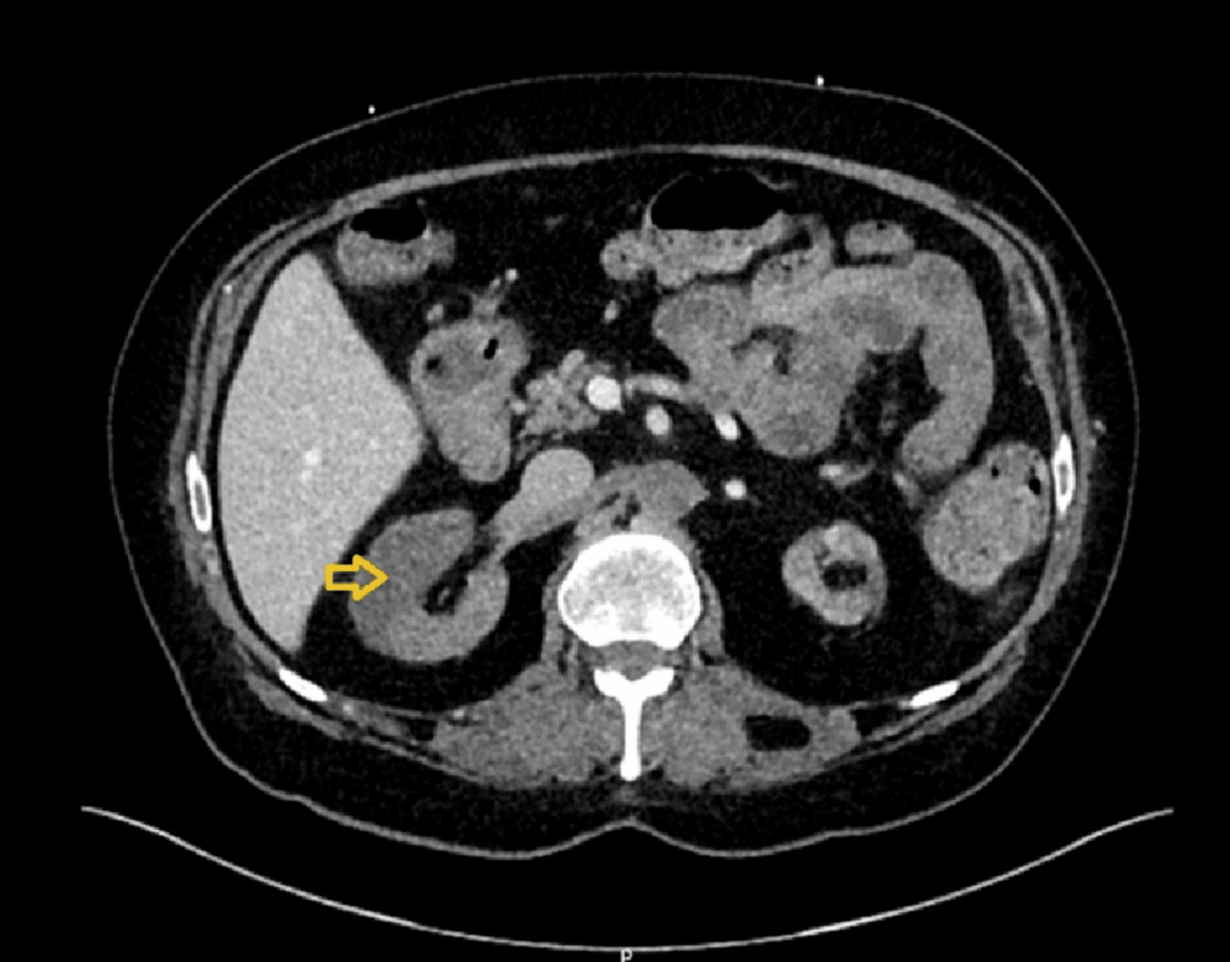
Right kidney infarct The above CT scan showed an acute infarction (yellow arrow) in the right kidney due to renal arterial occlusion.

Two days later, he began experiencing severe cramping abdominal pain accompanied by per rectal bleeding. Computed tomography (CT) scan revealed extensive thrombosis involving multiple vascular territories, including the abdominal aorta distal to the celiac axis with extension into the internal and external iliac arteries (Figure 3) and acute ischemia in a watershed area of the descending colon (Figure 4). There was acute infarction in the right kidney due to renal arterial occlusion (Figure 5), and the superior mesenteric artery (SMA) was also occluded. A thrombophilia screen was performed and returned negative, and the patient was not known to have any other significant prothrombotic state apart from cocaine use. However, the temporal relationship between ingestion of magic mushrooms and the acute onset of AKI with widespread thrombosis supports a precipitating role.

We initiated oral prednisolone 30 mg once daily for magic mushroom-associated myocarditis. He experienced recurrent episodes of per rectal bleeding and severe colicky abdominal pain. Intermittent dialysis was performed over a three-week period; however, he remained anuric, requiring regular dialysis (at least thrice a week), and continued to deteriorate clinically.

A multidisciplinary team (MDT) meeting was convened to review the ongoing management plan. Following thorough discussion, it was agreed that active intervention would no longer be appropriate given the overall clinical picture. Subsequently, the patient was referred to the palliative care team, with a shift in focus toward comfort measures and symptom control. The patient’s condition continued to decline, and he passed away peacefully.

## Discussion

Psilocybin, chemically designated as 4-phosphoryloxy-N,N-dimethyltryptamine (4-PO-DMT), is a naturally occurring tryptamine alkaloid present in more than 200 species of mushrooms, predominantly within the genera Psilocybe, Panaeolus, Conocybe, Gymnopilus, Stropharia, Pluteus, and Panaeolina [2]. For centuries, psilocybin has been used by Indigenous cultures as a tool for spiritual and ceremonial practices. In recent years, it has garnered significant attention in scientific research due to its potent psychoactive effects and potential therapeutic benefits.

Magic mushrooms produce their hallucinogenic effects primarily through psilocybin, which is rapidly metabolized into psilocin in the human body [3]. Psilocin, a serotonergic compound structurally similar to serotonin, acts as a partial agonist at several serotonin receptor subtypes, most notably the 5-HT2A receptor, resulting in altered activity in the prefrontal cortex and amygdala [4]. It is believed to induce euphoria, visual and auditory hallucinations, alterations in perception, a distorted sense of time, and perceived spiritual experiences [3,4]. Psilocin can also cause nausea, abdominal discomfort, and, in some cases, anxiety or panic attacks [2-4].

The simplest method is to consume dried or fresh mushrooms directly. However, their strong taste can be unpleasant, making them difficult to ingest. Many users brew magic mushrooms into tea by steeping them in hot water for 10 to 15 minutes, often adding herbs like ginger or lemon to enhance flavor and reduce the strong taste. Magic mushrooms can also be dried, ground into powder, and encapsulated for discreet, precise dosing. Alternatively, some people incorporate them into food, such as smoothies, chocolates, or other edibles, to mask their strong taste. This method allows for more controlled consumption while avoiding the unpleasant flavor of the mushrooms, making it a popular choice for many users.

Psilocybin mushrooms are generally considered to have a relatively low toxicity profile compared with other substances [2]. However, as with any substance, there are potential risks, especially when used irresponsibly or in combination with other drugs or alcohol [2]. Scientists have studied psilocybin and LSD for decades, revealing their promising potential to treat mental illnesses, especially substance addiction and depression [5].

Cocaine use has been linked to a wide range of vascular complications affecting both arterial and venous systems. Cocaine can enhance platelet aggregation and activation, promoting thrombus formation. It also increases circulating clotting factors such as fibrinogen while simultaneously reducing fibrinolysis, thereby contributing to a prothrombotic state. Cocaine also causes intense vasoconstriction, increasing the likelihood of thrombus formation [6,7]. Mushroom ingestion may have acted as a trigger in a vulnerable vascular system, resulting in the acute presentation. This synergistic effect aligns with reported cases in which recreational drugs with vasoconstrictive and prothrombotic properties interact to precipitate severe vascular events.

There are some case reports documenting AKI following the ingestion of magic mushrooms. In most instances, symptoms resolve with supportive treatment, and renal replacement therapy is not typically required [8]. However, in this particular case, the clinical picture was complicated by a history of cocaine use, contributing to the severity of renal impairment. As a result, renal replacement therapy was initiated with the expectation of eventual renal recovery. However, due to multiple complications arising from magic mushroom poisoning, the patient’s overall condition progressively worsened.

## Conclusions

This case illustrates the potential for severe multisystem toxicity resulting from psilocybin-containing "magic" mushrooms, particularly in the context of concomitant recreational substance use such as LSD or cocaine. The synergistic effects of polysubstance exposure can markedly amplify physiological stress, precipitating AKI and systemic complications that may necessitate renal replacement therapy. Clinicians should maintain a broad differential diagnosis for unexplained AKI, especially in younger individuals without pre-existing renal disease, and actively inquire about recreational drug use. Prompt recognition of toxin-related renal injury, supported by meticulous history-taking and corroborative information from collateral sources, facilitates timely therapeutic intervention and optimizes clinical outcomes.

In summary, this case report aims to raise clinician awareness of renal complications after magic mushroom ingestion, highlight diagnostic considerations to differentiate direct nephrotoxicity from alternative etiologies, and discuss implications for early recognition and management, emphasizing their impact on the clinical course and patient outcomes.
